# Assessment of invasive aquatic plant dynamics in the Lake Burullus wetland complex integrating remote sensing techniques

**DOI:** 10.1038/s41598-025-07892-9

**Published:** 2025-07-03

**Authors:** Muhammad A. El-Alfy, Hazem T. Abd El-Hamid, Hicham Ait Kacem, Amr E. Keshta

**Affiliations:** 1 National Institute of Oceanography and Fisheries, NIOF, Cairo, Egypt; 2 Department of Earth Sciences, Faculty of Sciences Ain Chock, University Hassan II of Casablanca, Km8 Route d’El Jadida, B.P. 5366, 20100 Maarif Casablanca, Morocco; 3 National Agency of Land Conservation of the Cadastre and Cartography, Rabat, Morocco; 4 Botany and Microbiology Department, College of Science, Tanta University, Tanta, 31512 Egypt; 5 Smithsonian Environmental Research Center, Edgewater, MD 21037 USA

**Keywords:** Satellite data, Alien species, Lakes, Statistics, Seasonal variations, Wetlands, Invasive species, Ecology, Wetlands ecology

## Abstract

The spread of invasive aquatic species in canals and wetlands poses significant challenges, including reduced water availability, disruption of native biodiversity, and obstruction of irrigation infrastructure. This study examines the distribution and environmental associations of two prominent invasive species *Pontederia crassipes* and *Pistia stratiotes* within the Lake Burullus wetland in Egypt. Field surveys were conducted to assess plant morphology and abundance, alongside measurements of water quality parameters including dissolved oxygen (DO), phosphate (PO_4_–P), ammonium (NH_4_–N), nitrite (NO_2_–N), nitrate (NO_3_–N), total nitrogen (TN), total phosphorus (TP), turbidity, and oxidizable organic matter (OOM). Remote sensing data, particularly the Normalized Difference Vegetation Index (NDVI), were used to monitor the spatial and seasonal dynamics of *Pontederia* in the Elshaklouba drain. The findings indicated that plant abundance was associated with specific water quality variables; however causality could not be determined due to the observational design of the study. NDVI analysis confirmed increased *Pontederia* densities during the summer months, consistent with field observations. The study also documented local management practices, primarily mechanical removal and the use of physical barriers, and briefly compared these with biological and integrated control strategies reported in recent literature. Additionally, the potential application of these species in phytoremediation and bioenergy applications is discussed, underscoring their dual role as both ecological threats and potential resources.

## Introduction

Coastal wetlands play a vital role in supporting both human populations and biodiversity, offering numerous benefits such as flood control, storm protection, and climate regulation^1^. However, these ecosystems face increasing pressure from invasive alien plant species (IAPS), which disrupt ecological processes and degrade habitat quality. This issue is particularly evident in the Burullus wetland complex in the Egypt’s Nile Delta, where aquatic invasive species like *Pontederia crassipes* and *Pistia stratiotes* threaten the sustainability of these wetland ecosystems. According to the National Invasive Species Council^2^, the IAPSare defined as, “an alien species whose introduction does or is likely to cause economic or environmental harm or harm to human health.” These species can significantly impact local biodiversity, impair ecosystem services, and reduce environmental quality. They disrupt natural water cycles, lower water availability, and deteriorate water quality by increasing soil erosion, altering nutrient dynamics, and contributing to excessive nutrient accumulation^3–6^. Despite these considerable impacts, there is a lack of detailed researches on the specific effects of invasive species on water quality, particularly studies that incorporate advanced monitoring tools and techniques.

Invasive species can worsen the impacts of water pollution by altering the physical and chemical properties of the ecosystem they invade. For instance, some invasive aquatic plants can change sediment composition and water quality, impeding the growth of native plants and disrupting local food webs^7^. Moreover, some invasive species may introduce new parasites, stressing native populations already weakened by pollution^7^. This dynamic not only threatens biodiversity but also compromises the health of ecosystems that provide essential services, such as water filtration and habitat stability. The interplay between invasive species and pollution underscores the need for integrated management strategies that address both issues simultaneously.

Invasive species can negatively impact the environment, economy and human health, so the early monitoring and detection are very significant. Traditional field survey methods may be replaced or integrated with more efficient and cheaper methods such as remote sensing methods^8^. Many authors used Landsat data for detection water hyacinth in aquatic systems using vegetation spectral indices in the detection process^9,10^. Invasive species not only impact human health but also disrupt natural physical processes. They can alter the amount and timing of runoff, increase erosion and sedimentation in addition to influence on water availability^11^. Invasive species can disrupt the nitrogen. The disruption significantly impacts essential algae and higher plants, altering the structure of the food web by changing nitrogen absorption^12^. Any alteration in nitrogen levels in water body affect negatively on ecosystem as degradation of water quality.

While the ecological impacts of *Pontederia crassipes* and *Pistia stratiotes* are well-documented globally, there remains a significant gap in understanding their spatial–temporal dynamics within the Lake Burullus wetland, a Ramsar site of international importance. Previous studies have largely focused on field-based assessments or coarse-resolution imagery, limiting the ability to monitor rapid vegetation changes. This study addresses this gap by integrating high-resolution Landsat OLI8 images with advanced vegetation indices to map and analyze the spread of invasive aquatic plants over time. To our knowledge, this is the first study to apply such a remote sensing-based approach in Lake Burullus, offering new insights into the potential of satellite data for wetland management and conservation.

The main objectives of this study were to assess the impact of invasive aquatic plants, specifically *Pontederia crassipes* and *Pistia stratiotes*, on the ecological dynamics of the Burullus wetland complex under varying salinity and nutrient conditions. A particular focus was placed on examining the behavior and distribution of invasive species, especially *Pontederia*within the study area and its relationship to key water quality parameters and distribution, using Landsat satellite data. The Elshaklouba drain served as a case study for applying remote sensing data. Furthermore, the study aimed to integrate remote sensing with environmental indicators to monitor and assess the spatial distribution and density of invasive species, particularly *Pontederia*, across Burullus Lake. By analyzing spatial and environmental data, this study sought to identify the main factors driving the spread of these species, evaluate their ecological impacts, and provide insights for effective management strategies to mitigate their effects on the wetland ecosystem.

## Materials and methods

### Study area

**Fig. 1:**
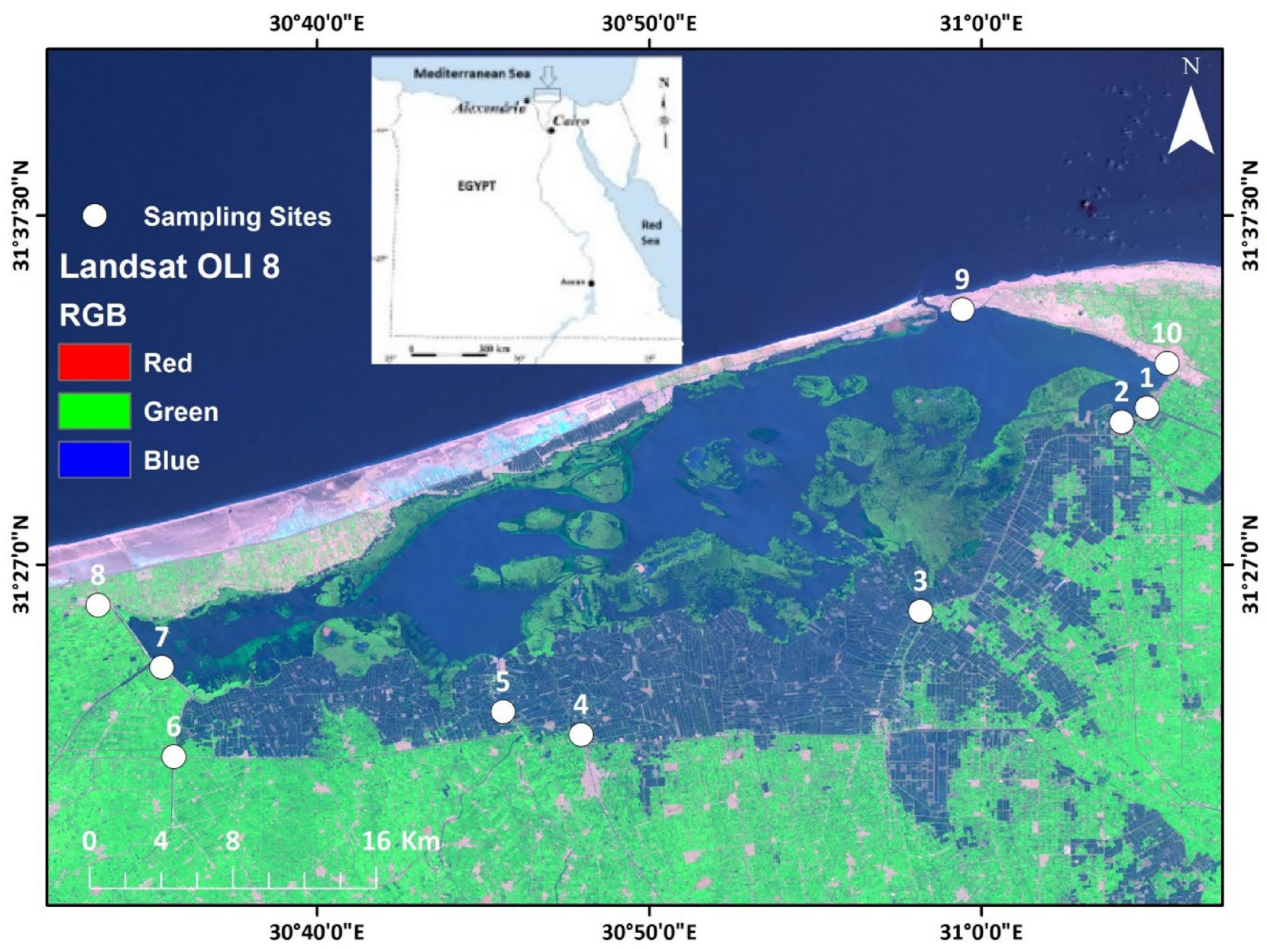
Satellite imagery for the study site map depicts Lake Burullus with sampling locations marked in white (labeled 1–10) in red color. The inset gray canvas map on the top left is for Egypt. Map produced by ArcGIS Desktop 10.5.

**Fig. 2:**
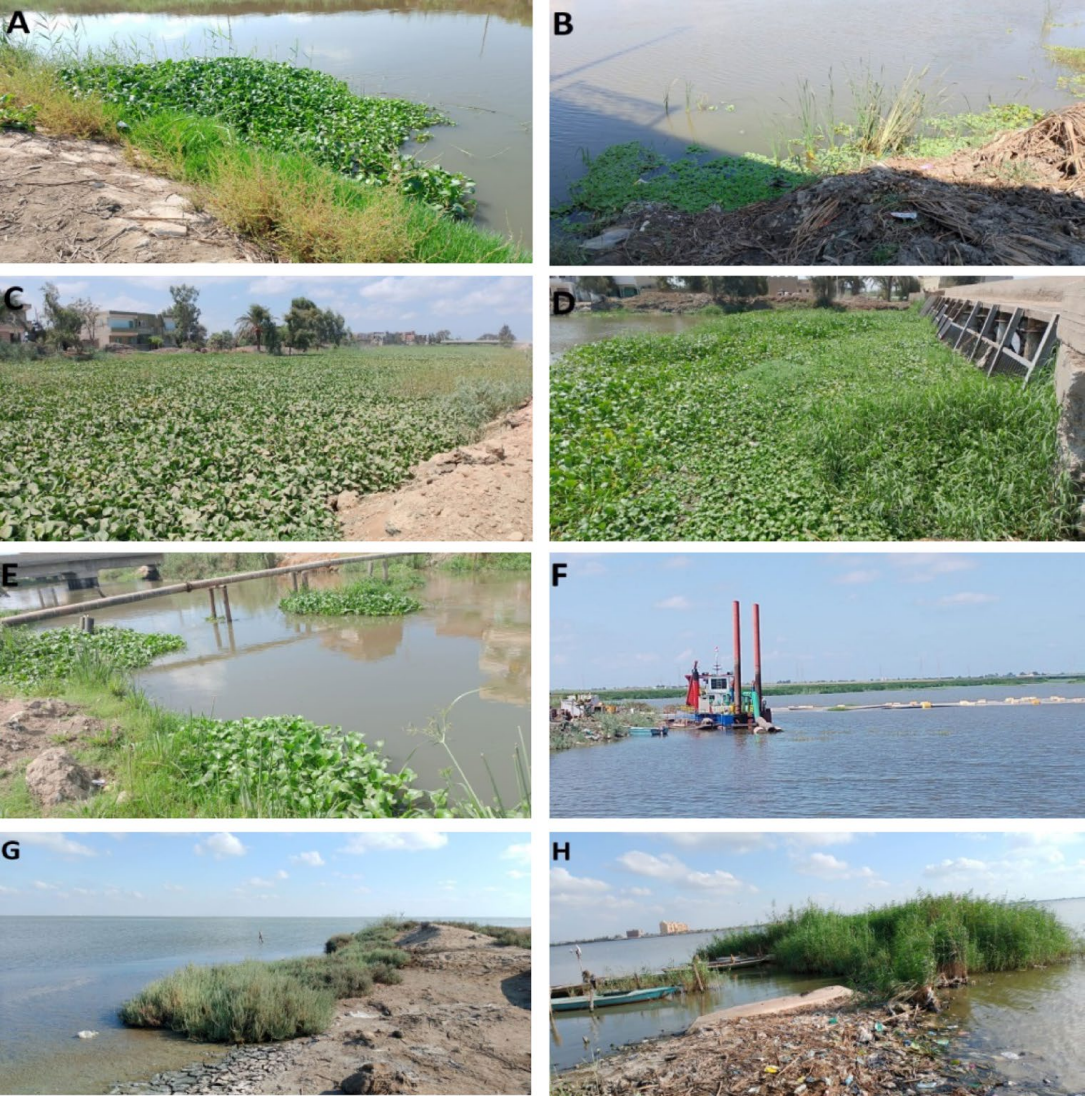
Onsite field images showing the distribution of invasive alien plants along the Burullus wetland. (**A**) Tirra drain, (**B**) Drain 7 indicating presence of *P. stratioes* beside *E. crassipes,* (**C**) Dense presence of *Pontederia,* (**D**) Dense presence front of Elkobry elaloy at Elshaklouba, (**E**) Elhoks station, (**F**) Digging practice in front of Brinbal outlet, (**G**) Halophytes species nearby Elboughaz and (**H**) beside Cornish Baltim city. All field images were captured during the field trip.

The study area is located in the central part of the Nile Delta and encompasses the Burullus wetland complex. It lies between longitudes 30°30′ and 31°10′E and latitudes 31°19′ and 31°36′N. It is known as a brackish water wetland complex with a mean depth of 115 cm^13^. This wetland receives drainage waters from eight main drains, listed from east to west: East Elburullus, Elkashaa, Tirra, Drain 7, Damru, Elshaklouba, and Elhoks. A freshwater canal, known as the Brinbal canal, is located at the western part of the lake. Another main drain, the Kitchener drain, does not discharge its water directly into the wetland but rather through lateral drains, with water reaching the lake via the Elkashaa drain^14^. The Burullus wetland complex is characterized by its variability in formations and biodiversity^15,16^. Sampling sites were distributed across the Burullus wetland complex, as illustrated in Fig. 1. Field photographs illustrating invasive alien aquatic plant species within the study area are presented in Fig. 2.

### Collection of water samples

Water samples were collected on September 3, 2024, from the Burullus wetland complex, focusing on the drainage network, to monitor the floating invasive aquatic species *Pontederia crassipes* and *Pistia stratiotes*. At the time of sampling, the plants were widespread throughout the lake, especially in the southern areas. The collected water samples were transported to the laboratory at Baltim station of the National Institute of Oceanography and Fisheries, NIOF, Egypt for further analysis. The samples were filtered using 0.45 µm membrane filters for nutrient analysis. Ammonium ions in the samples were fixed in the field^17^.

### Analyses of water parameters

The methodology employed in this study is summarized in Table 1. Several water parameters were analyzed to assess the environmental conditions influencing the presence and distribution of invasive plants, including temperature (T°C), pH, salinity, nutrients, chlorophyll a, oxidizable organic matter (OOM), and dissolved oxygen (DO).

**Table 1:** Water quality parameters analyses with methodology and formula.

Water parameters	Instrument & methodology & formula
Temperature (T°C)	Thermometer
Hydrogen ion concentration (pH)	pH-meter
Salinity ‰	Salinity-meter
Turbidity NTU	Turbidimeter
DO mgL^−1^	Winkler method^18^
OOM mgL^−1^	Permanganate oxidation method^19^
	$$\text{OOM}=\frac{{\text{V}}_{\text{blank}}-{\text{V}}_{\text{sample}}*8000*{\text{N}}_{{\text{Na}}_{2}{\text{S}}_{2}{\text{O}}_{3}}}{{\text{V}}_{\text{sample}}}$$
Chlorophyll (a) µgL^−1^	the score UNESCO Eq.^20^ [(11.64 * E663)−(2.16 * E645) + (0.1 * E630)] *10 / V
Nutrients (NH_4_, NO_2_, NO_3_, PO_4_, TN & TP) µgL^−1^	Developed colors were determined spectrophotometrically and results expressed in µgL^−1^^17^

### Environmental characteristics and impacts of two invasive aquatic plants

*Pontederia crassipes*(commonly known as Water hyacinth) is a major macrophyte that disrupts water resources in many countries, potentially causing significant economic losses due to its rapid spread^21^. It is listed among the world’s 100 worst invasive alien species^22^. The morphological characteristics of *P. crassipes* vary with growth conditions. Petioles are elongated with circular leaves in dense stands and short with kidney-shaped leaves in less dense mats^23^. It belongs to the family Pontederiaceae and is an effective phytoremediator for metal ions such as cadmium (Cd) and chromium (Cr)^24^.

*Pistia stratiotes* belongs to the Araceae family, is a perennial monocotyledon with thick, soft leaves. This floating macrophyte is found on the water surface. The roots extend directly beneath the base of the floating leaves and emit a distinct musty odor^25,26^. The morphological characteristics of *Pistia stratiotes* are influenced by salinity, which can reduce leaf width. Conversely, increases in organic carbon and nutrient ions enhance plant biomass^27^. It thrives in slow-moving water habitats such as canals, ponds, and lakes^28^.

### Measurements of plant parts

Specimens of *Pontederia crassipes* and *Pistia stratiotes* were collected in the field. The tested species were selected from the most abundant mats. The length of the petioles and stolons, as well as the height and width of the leaves, was measured. Measurements were taken from the minimum and maximum dimensions of *Pontederia* plants^29^.

### Remote sensing data collection

Landsat OLI8 images were obtained from the Earth Explorer website and pre-processed using radiometric calibration in ENVI 5.3^30^. The selected images acquisition dates were: 26 November 2023, 01 March 2024, 02 April 2024, 13 June 2024, 08 August 2024, 10 September 2024 and 03 October 2024. The spatial resolution of image bands was 30 m. These images were used assess the density of vegetation cover along the drain, particularly the invasive alien water hyacinth, which is highly distributed in the drain water. NDVI density detection was performed using ArcGIS 10.5 to calculate the area covered by vegetation. The NDVI classes were categorized into four levels based on vegetation density^31^ (Table 2). This analysis was based on the methodology of Mucheye et al.^10^ for water hyacinth detection, using the following equation:

**Table 2:** Values and categories of NDVI.

NDVI value	Vegetation category
0–0.01	No density
0.01–0.30	Low density
0.31–0.60	Moderate density
0.60–1	High density

$$\text{NDVI}= {\text{Band}}_{5}-{\text{Band}}_{4}/{\text{Band}}_{5}+{\text{Band}}_{4}$$

According to Mucheye et al.^10^, water hyacinth is associated with NDVI values ranged between 0.6 and 0.95.

### Statistical analysis

To assess the relationship between water quality and invasive plants in the study area, Principal Component Analysis (PCA) was employed as an optimization technique to highlight correlations and extract key features. Additionally, a linear regression model, based on correlation analysis, was used to estimate vegetation density as an indicator of invasive plant presence.

## Results and discussion

### Human activities and water quality roles in distribution of invasive plants

**Fig. 3:**
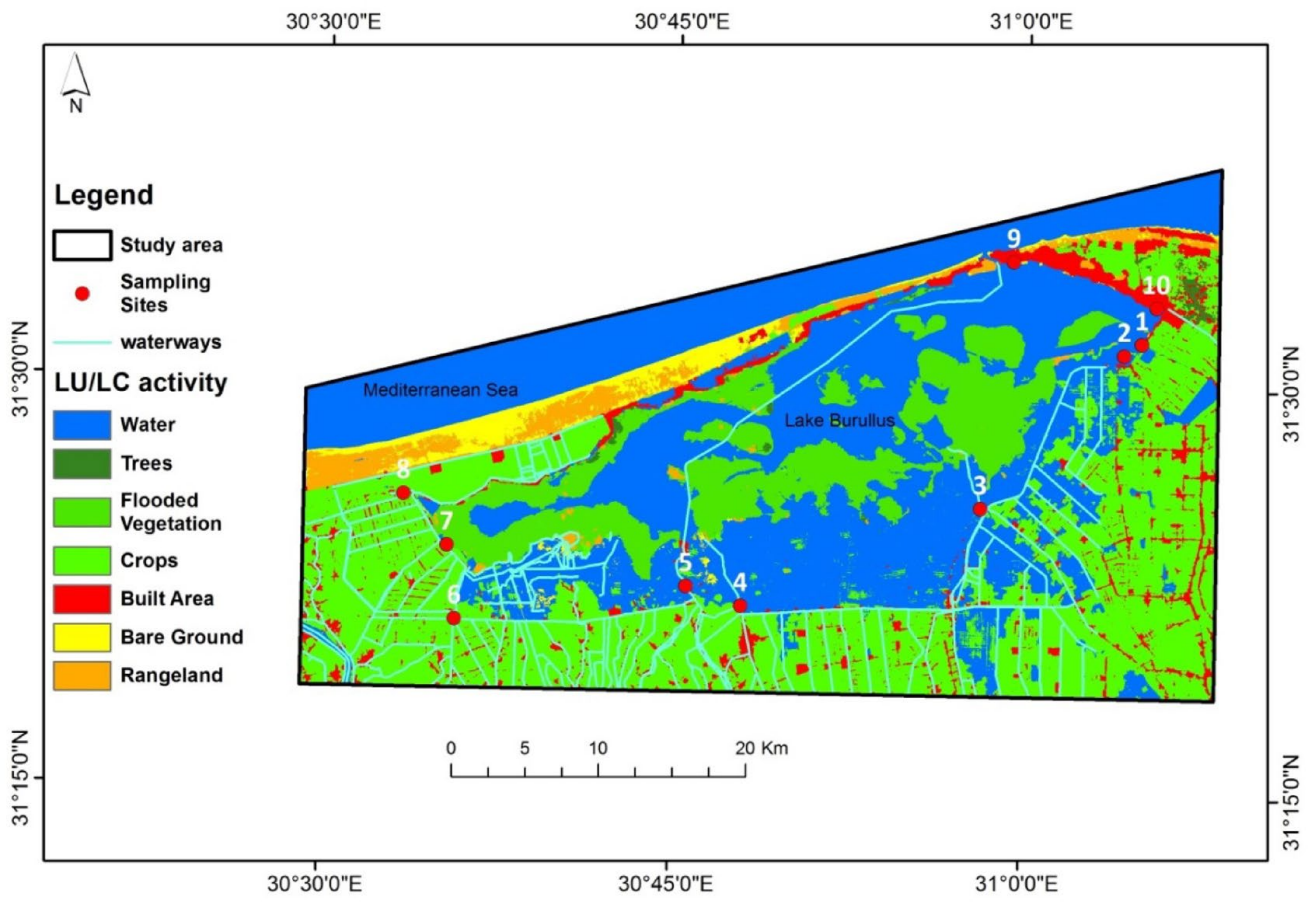
Land use/cover map extracted using ArcGIS 10.5 (*Source* sentinel 2 10m land use/cover map time series of the world produced by impact observatory and ESRI).

In the study area, various human activities surrounding and within the vicinity of Lake Burullus were identified including agricultural, industrial, fish farms, and urban areas^32^. These activities effect badly on water quality. Peters et al.^33^ mentioned many water quality issues which were related to anthropogenic activities namely; organic matters, metal ions, nutrients especially nitrogen and phosphorus, pesticides, herbicides, exotic and invasive species and others. Understanding these activities is essential to evaluating its impacts. Most of studied drains were characterized by agricultural wastes with the exception for Elhox drain (site 6) which receives industrial wastes. In addition, no invasive aquatic plants were recoreded at Elboughaz area (site 9). Similarly, the site of Baltim (site 10) lacked the presence of these two plant species^32,34^. A sentinel 2 10m land use/cover map was implemented to indicate different activities around the sampling sites as illustrated in Fig. 3.

Based on field observations, invasive aquatic plant species such as *Pontederia* and *Pistia* were absent in areas with elevated salinity levels. This pattern is consistent with previous researches; for instance, Haller et al.^35^ reported that *Pistia* exhibits low salinity tolerance and does not survive in water with salinity exceeding 2.5 ppt. In our study, *Pistia* was observed only in Drain 7 (Table 3), where salinity was relatively low (1 ppt; Table 4). Similarly, *Pontederia crassipes* (water hyacinth) is known to be sensitive to salinity, with higher levels inhibiting its growth and potentially leading to mortality^36^. While our findings align with these reports, it is important to note that the absence of these species in salt-impacted areas may be influenced by multiple interacting factors, and salinity alone cannot be confirmed as the sole determinant.

**Table 3:** Measurements of plant parts along the studied sites Nd: non detected, *P.c.*: *Pontederia crassipes and Ps: Pistia stratiotes.*

Site nos.	Name	Coordinates		Species	Plant parts in cm						
		N	E		Leaves				Petioles		Stolon
					Width		Height				
					Min	Max	Min	Max	Min	Max	
1	Elkashaa	31.5523	31.0931	*P.c.*	2.5	6.9	2.5	6.5	7.5	10	16.5
2	Tirra	31.5214	31.0702	*P.c.*	6.5	11	7	11	16	28	15.5
3	Drain 7	31.4265	30.9692	*P.s.*	5.5	8.5	–	4			
				*P.c.*	8	11	7.5	11	20	33	20
4	Damru	31.3645	30.7990	*P.c.*	6.5	12	7	11.5	14.5	30.5	14.5
5	Elshaklouba	31.3761	30.7598	*P.c.*	9	13	12	14	24.5	56	NA
6	Elhoks	31.3534	30.5948	*P.c.*	2.5	12.5	3	10.5	13	23	12.5
7	Brinbal	31.4560	30.6313	*P.c.*	4.5	6	4	6.5	5.5	16	NA
8	West Elburullus	31.3715	30.5924	*P.c.*	5	10.5	5	8	8.5	15	14
9	Elboughaz	31.5806	30.9902	ND	ND	ND	ND	ND	ND	ND	ND
10	Baltim	31.5524	31.0900	ND	ND	ND	ND	ND	ND	ND	ND

**Table 4:** Values of water parameters within sites of sampling.

Site nos.	T °C	pH	Turbidity NTU	Sal. ‰	DO	OOM	Chl_a	NH_4_	NO_2_	NO_3_	TN	PO_4_	TP
					mgL^−1^		µgL^−1^						
1	31	8.95	59	3	4	2	543.32	177	104.48	339.46	2662.94	8.58	162.75
2	30	8.35	80	2	2.5	9.6	234.84	3690	146.39	431.53	5061.84	43.95	362.39
3	29	8.36	66	1	2.7	11.2	437.40	2726	123.87	447.53	3010.70	22.05	454.62
4	31	7.91	14.2	3	3.2	6.4	317.07	2562	113.55	273.91	3657.92	62.49	506.70
5	32	8.15	19	1	2.4	12.8	89.89	2743	49.422	424.21	3748.08	89.59	690.06
6	30	8.32	37	1	1.2	9.6	33.16	1587	130.12	502.83	4388.86	65.40	424.24
7	32	8.79	26	4	10	8	56.28	406	31.59	176.98	3355.24	15.47	371.07
8	30	8.07	64	2	2.2	7.2	3.54	1303	127	771.02	4105.50	35.38	336.35
9	31	8.61	25	15	7.5	10.4	182.46	1443	34.72	281.03	5106.92	17.31	298.38
10	29	8.14	152	5	4.5	1.2	395.14	868	314.99	609.10	5412.82	16.23	230.02

Water hyacinth has been associated with reduced phytoplankton productivity, likely due to its dense mats which can deplete dissolved oxygen and limit light penetration^37,38^. In our study, *Pontederia* distribution appeared to coincide with specific physicochemical conditions, including moderate nutrient levels, pH, and turbidity. For example, the highest *Pontederia* abundance was recorded at Elshaklouba, where turbidity was low and both water temperature and pH fell within the species’ reported optimal ranges^39–41^. While these associations suggest favorable conditions for *Pontederia*, we caution against inferring direct causality, as other unmeasured environmental or biological factors may also contribute.

Previous studies have emphasized the potential of *Pontederia* and *Pistia* in phytoremediation^42^. In our study area, sites with higher plant abundance also exhibited lower levels of oxidizable organic matter (OOM) and nutrients, which may reflect the plants’ capacity to absorb or retain pollutants. However, these patterns are correlative, and further experimental or longitudinal studies are needed to confirm the extent of their remediation effects. Conversely, dense *Pistia* mats may reduce wind-induced mixing, potentially leading to thermal stratification, lower oxygen levels, and increased nutrient concentrations^43–45^. These findings underscore the complex and context-dependent roles of invasive aquatic plants in shaping water quality.

Nutrient levels are a key factor influencing the invasion of water hyacinth^46^. Previous studies noted that water hyacinth is highly productive with increased nitrogen levels, although it can grow at low levels. This aligns with our findings, as all sites with high nitrogen levels exhibited *Pontederia* presence and expansion, except those with high salinity. *Pontederia* varies in height from a few centimeters to nearly a meter, with leaves typically 15–20 cm in length and width^47^. Plant size and morphology are influenced by nutrient concentrations, particularly phosphorus (P) and nitrogen (N)^48^. In this study, *Pontederia* morphological characteristics varied across locations, potentially due to nutrient availability. The maximum leaf height and width were recorded at Elshaklouba, ranging from 13–14 cm, with a maximum petiole length of 56 cm. The lowest maximum leaf height and width were observed at Elkashaa and Brinbal, with values of 6.5–6.5 cm and 6–6.5 cm, respectively (Table 3). Ismail et al.^49^ reported that floating macrophytes thrive on the water surface under favorable water quality conditions, with phenotypic plasticity aiding their adaptation. Submerged species are more common in shallow water bodies. Floating aquatic macrophytes can be used to remove or reduce nutrient loads from wetland water, mitigating the spread of other invasive aquatic weeds. Species like *Pontederia* can adsorb significant amounts of nutrients^50^.

### Management processes

In the study area, two primary management practices were identified for controlling invasive aquatic plants. The first involves mechanical removal typically using excavators to clear drains, lagoons, and streams from unwanted plants^51^. The removed plant biomass is typically deposited on canal banks to dry (Wade, 1990). The second practice includes establishing of nets across drainage channels to prevent the spread of invasive plants into lake waters. While these mechanical approaches offer immediate results, they are often labor-intensive, costly, and require repeated application. As Tang^52^ and Khan et al.^53,54^ have noted, more sustainable and cost-effective strategies are needed for long-term control.

In contrast, biological control methods such as the introduction of host-specific insects or pathogens have shown promise in reducing invasive plant populations over time with minimal environmental disruption. However, they may take longer to establish and require careful ecological assessment. Recent studies advocate for integrated management approaches that combine mechanical removal with biological agents, offering a more balanced and effective solution by leveraging the strengths of each method. Despite these advancements, many regions, including South Africa, Lake Mutirikwi in Zimbabwe, and sites around Lake Victoria in Ethiopia, continue to rely primarily on mechanical or physical methods^54^, underscoring the need for broader adoption of integrated strategies.

**Fig. 4:**
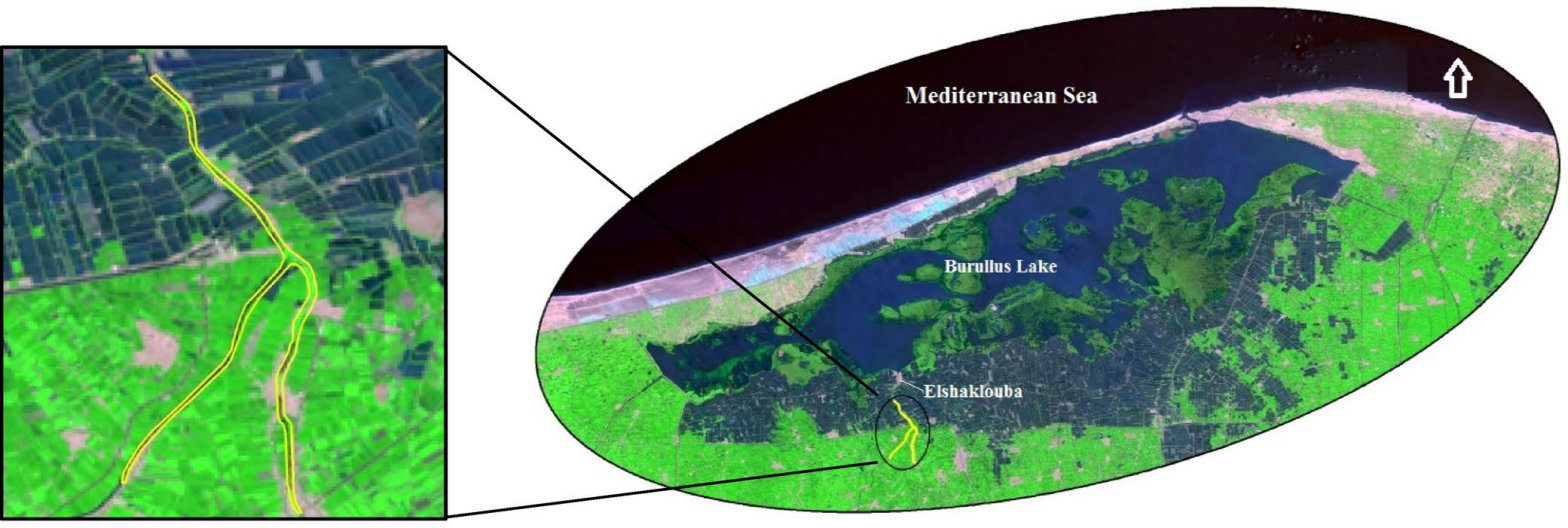
Case study of Elshaklouba drain to observe distribution of *Pontederia Crassipes.* *Source* Landsat image from https://earthexplorer.usgs.gov/ and shape file produces using ArcGIS 10.5.

**Fig. 5:**
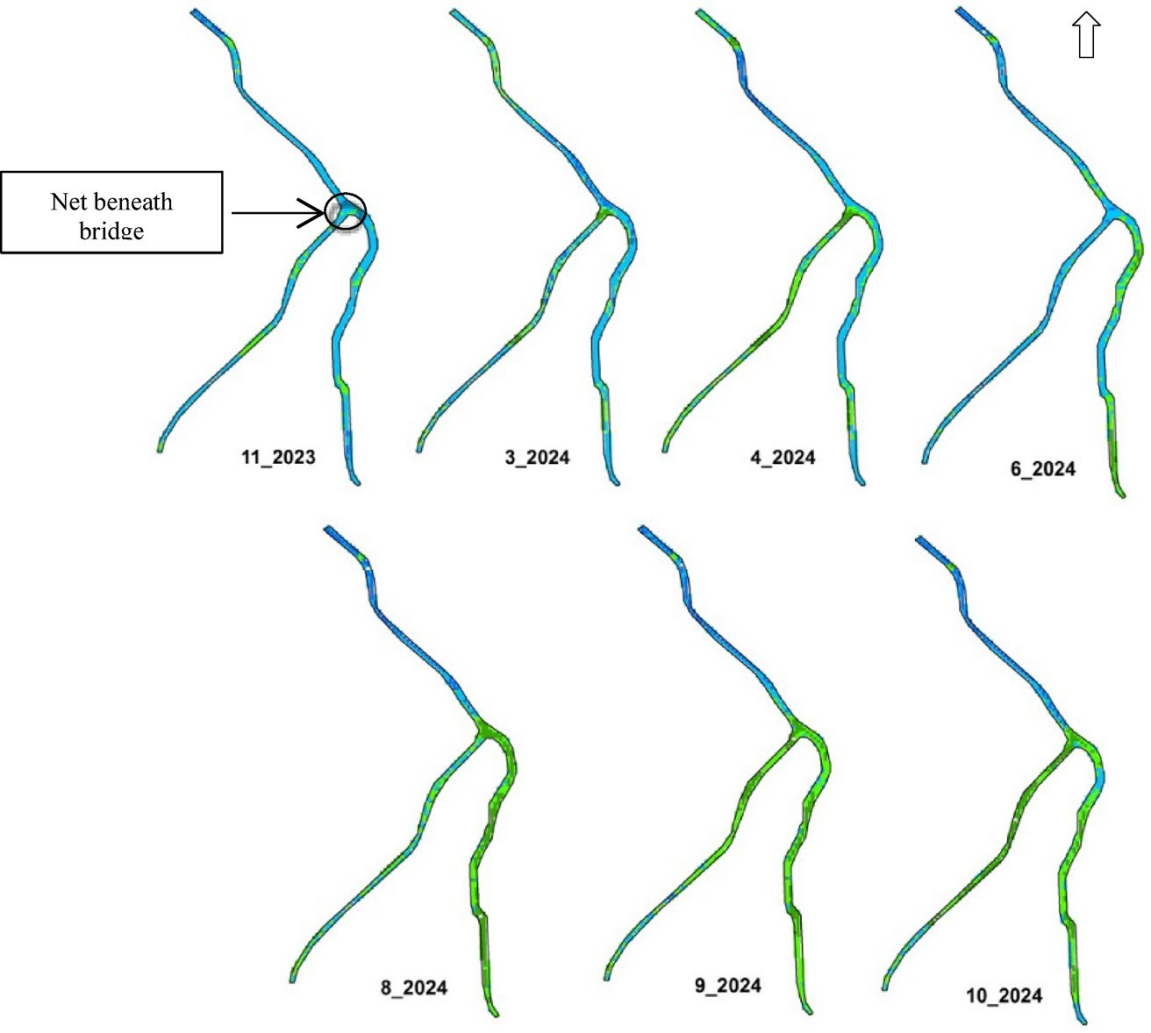
Distribution maps of *Pontederia* mats along Elshaklouba drain from values (NDVI = 0.6–0.95) using ArcGIS 10.5.

The Elshaklouba drain site was selected for monitoring *Pontederia* invasive species using Landsat OLI8 satellite images, as illustrated in Fig. 4. It is located in the southwestern part of the lake. According to the NDVI categorization, there were four classes: no vegetation, low-density vegetation, moderate-density vegetation, and high-density vegetation. Based on Mucheye et al.^10^, water hyacinth spread was concentrated within NDVI values of 0.6–0.95. High-density vegetation along the drain was observed during the summer season. This result aligns with Wirngo et al.^55^, who found that water hyacinth was present in the Wouri River throughout the year and was more prevalent during the dry season than the rainy season. NDVI density area values are shown in Table 5 and Fig. 5.

**Table 5:** Values of vegetation density and expected area coverage by *Pontederia crassipes* mats along Elshaklouba drain.

Date	Season	Vegetation density area (m^2^)			
		No	Slight	Moderate	High
26-11-2023	Autumn	17.1	411.3	62.1	ND
01-03-2024	Winter	40.5	276.3	126	46.8
02-04-2024	Spring	32.4	252	207	ND
13-06-2024	Spring	37.8	322.2	104.4	26.1
08-08-2024	Summer	58.5	136.8	155.7	138.6
10-09-2024	Summer	48.6	118.8	229.5	92.7
03-10-2024	Autumn	59.4	140.4	168.3	122.4

**Fig. 6:**
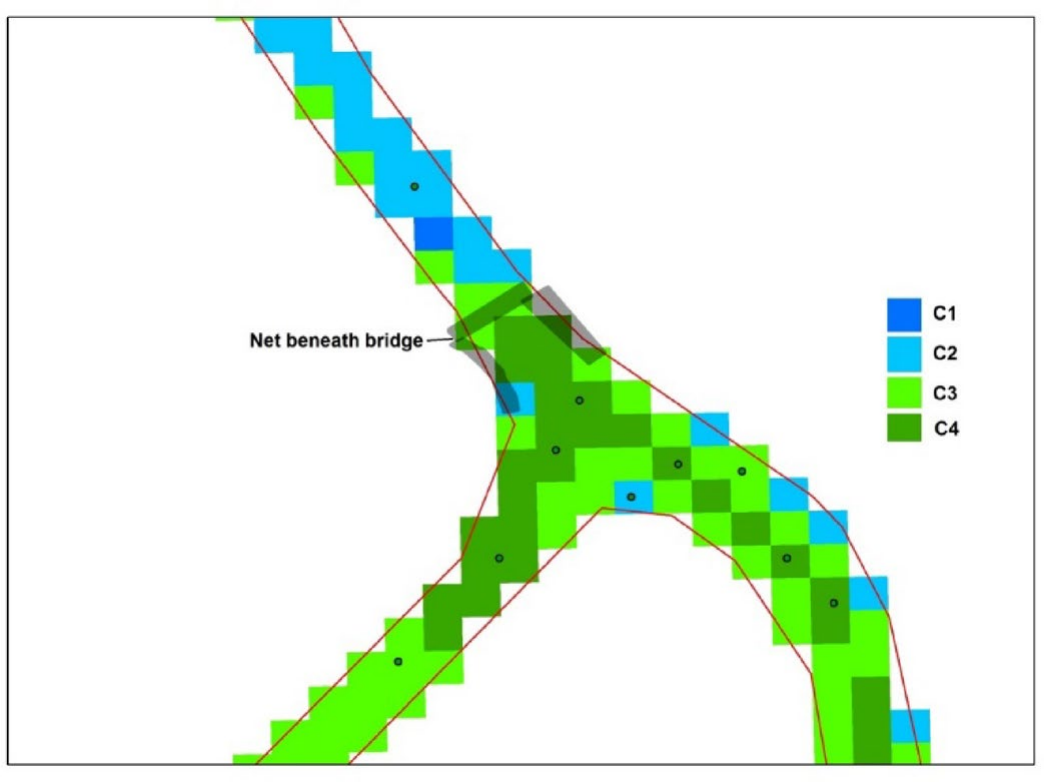
Pixel values of plant cover according to reclassifying and field observation using ArcGIS 10.5

Reclassification was applied as follows: C1 (< 0.01) represented non-vegetative surfaces such as water, soil, urban areas, and bare lands; C2 (0.01–0.3) indicated low vegetation cover; C3 (0.31–0.60) represented moderate-density vegetation; and C4 (> 0.61) indicated high-density vegetation. Class 4 is most indicative of water hyacinth presence. It is probable that class 4 represents high-density *Pontederia* mats, while sparse *Pontederia* may fall within class 3. Another interpretation is that older, denser *Pontederia* may not reflect as strongly in the green bands as younger, more nutrient-rich plants. One limitation of this study is the reliance of multispectral imagery to detect and observe invasive plant species along the lakes. Additionally, interference from other plant reflectance may impact the results. Validation was conducted using random sampling along the drain and field observations. Pixels where *Pontederia* mats were densely distributed corresponded to values equal to or greater than 0.7, while moderate-density mats had values lower than 0.6 (Fig. 6).

### Statistical analysis

#### PCA analysis

**Fig. 7:**
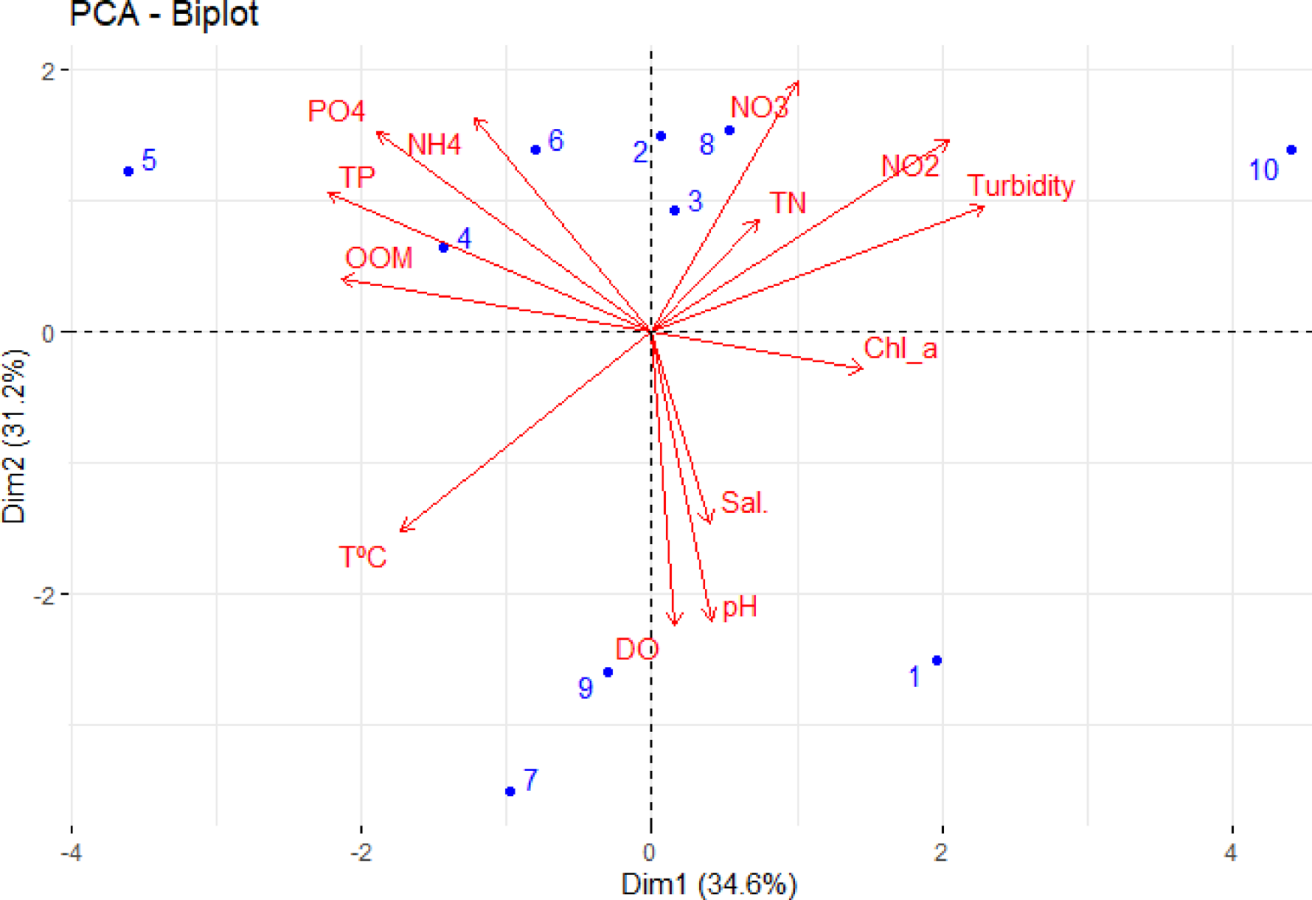
Principle component analysis (PCA) of water quality and sampling sites.

Based on Principal Component Analysis (PCA) (as shown in Fig. 7), salinity and dissolved oxygen (DO) are the most influential factors explaining the environmental variation across the study area. Invasive floating plants, such as *Pontederia crassipes*, reduce light and oxygen, leading to fish kills and negatively impacting the aquatic ecosystem^38^. PCA is widely recognized as an effective multivariate tool for identifying sources of pollutants along the study area. It simplifies complex datasets by converting them into a set of new components, where each component represents a cluster of interrelated variables^56^. Data showed that the first two components; PCA1 and PCA2 explain about 66% of the total variance in the dataset. Site 10 (Baltim) is associated with higher nutrient levels and turbidity which indicating poor quality; these may be attributed to high agricultural wastes along this site. El-Mezayen and Abd El-Hamid^57^ mentioned that high nutrients levels are attributed to an increase quantity of fertilizers from agricultural wastes. Despite of recent improvement of Buruulus Lake, it suffers from high content of nutrients that increase water pollution^58^.

On the other hand, sites 7 and 9 are associated with high levels of DO. Finally, PCA is an effective tool to distinguish among different sites along the study area. PCA data showed that sites; Drain 7 and West Elburullus have the same properties with high total nitrogen, nitrate, nitrite and turbidity. These may be attributed to discharge of different wastes. Elkashaa (site 1) is associated to chl_a, pH and salinity as shown from PCA analysis. These are related to high effluent to this drain from mixed different pollutants sources. These finding reflect the impact of human activities such as agricultural runoff, indusial dishrags and urban wastes on the water quality of Burullus Lake.

#### Correlation analysis

**Fig. 8:**
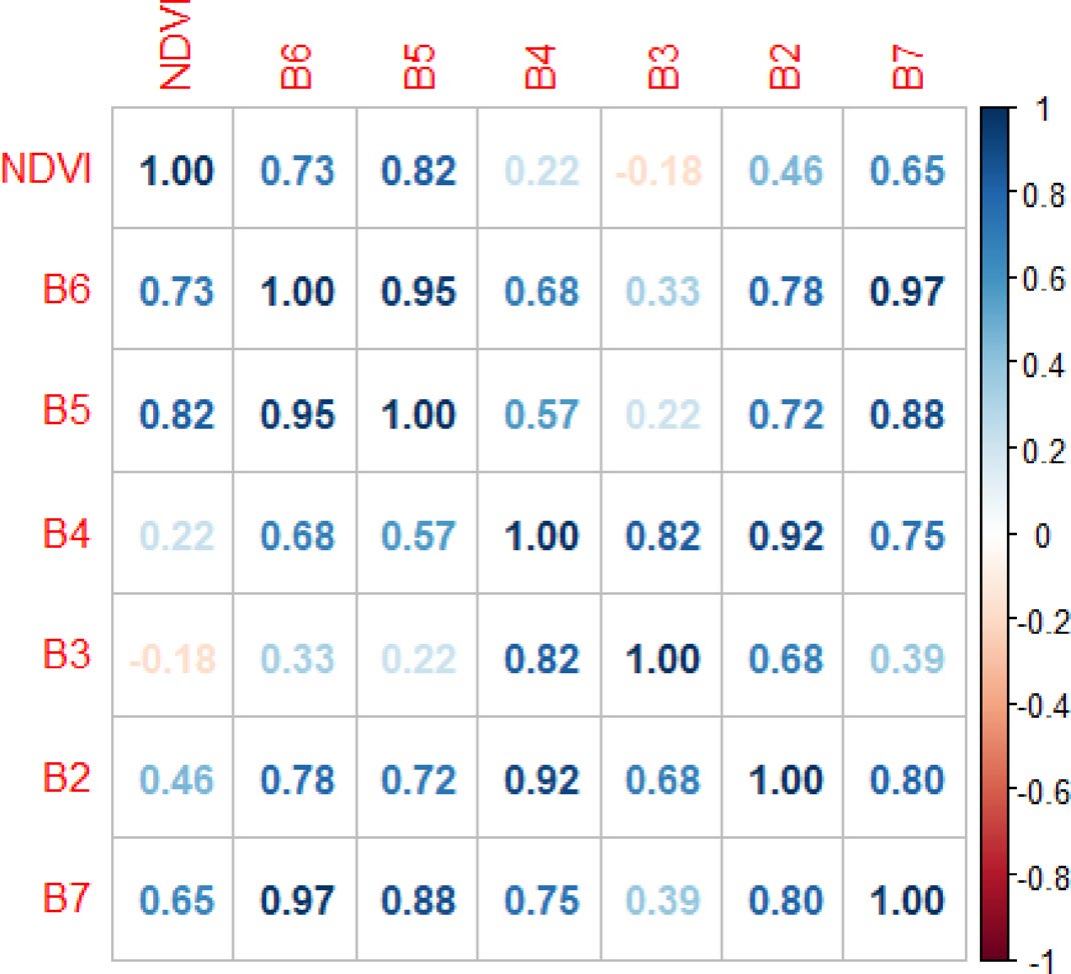
Correlation analysis of remote sensing data.

Data indicated that NDVI has a strong positive correlation with bands B5, B6, and B7, with R^2^ values of 0.82, 0.73, and 0.65, respectively, as shown in Fig. 8. Invasive plants can be identified using NDVI and other spectral indices. Evaluating the performance of multiple indices beyond the commonly used ones is crucial for mapping and modeling invasive species distribution^59^. Iqbal et al.^60^ stated that invasive plants exhibit a significant spectral signature in the shortwave infrared (SWIR) band due to their influence on soil and vegetation moisture. Furthermore, numerous studies have demonstrated a strong relationship between NDVI and the near-infrared (NIR) band.

#### Regression analysis for invasive species coverage

**Fig. 9:**
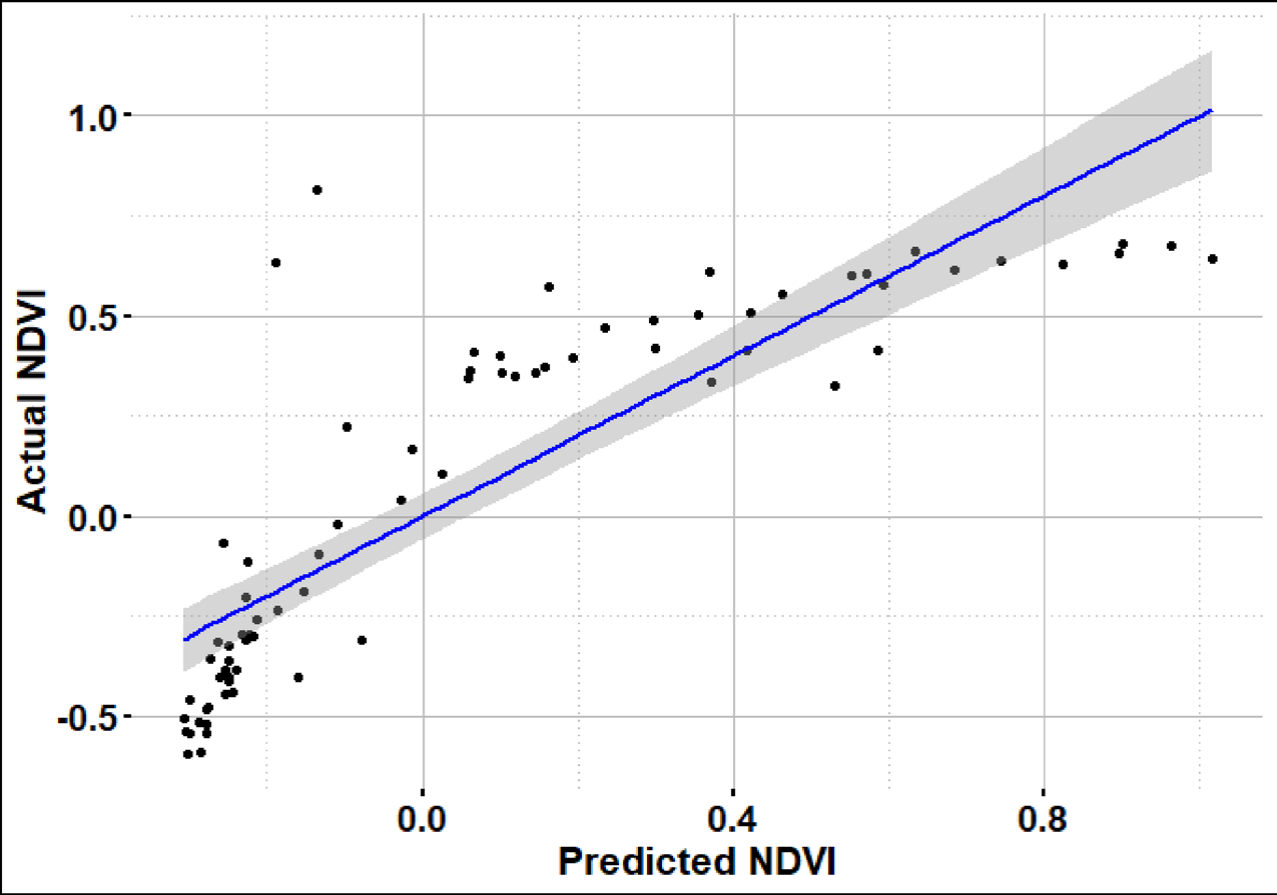
Multi regression model of invasive species based on NDVI.

Bands as B5, B6, and B7 were selected as indication to invasive plants due to their strong correlation with NDVI. A regression model for invasive plants as an NDVI indicator was applied, yielding an R^2^ value of 0.71, as shown in Fig. 9. The resulting equation can be used to identify or map the distribution of invasive plants using NDVI and their reflectance in the near-infrared (NIR) and shortwave infrared (SWIR) bands.

$$\text{NDVI}=-0.34+8.61*{\text{B}}_{5}-6.84*{\text{B}}_{6}+2.16*{\text{B}}_{7}$$

## Conclusions

This research highlighted that *Pistia* cannot tolerate salinity levels above 2.5 ppt, resulting in its absence in saline areas. Conversely, *Pontederia* thrived in low turbidity conditions, with higher abundance at sites like Elshaklouba. Both species effectively reduced total organic matter and nutrient levels, thereby improving water quality. Additionally, morphological variations in *Pontederia* were linked to nutrient concentrations, showcasing their adaptability. Overall, these findings suggest the potential for using these species in phytoremediation efforts in affected ecosystems. The distribution of invasive aquatic plants along the Burullus wetland is influenced by multiple factors, including nutrient concentrations, salinity levels, and human activities.

The integration of remote sensing data with field observations proved to be an effective approach for tracking the seasonal spread of invasive aquatic species species. In particular, *Pontederia crassipes* and *Pistia stratiotes* demonstrated a strong capacity to absorb pollutants such as phosphate, nitrogen compounds, and oxidizable organic matter, indicating their potential use in phytoremediation. Conversely, their removal can enhance water quality by increasing dissolved oxygen levels and improving light penetration, which benefits aquatic ecosystems. However, the study faced limitations; while NDVI provided useful insights, the use of spectral signatures would have offered greater accuracy in detecting plant distribution patterns.

## Data Availability

The authors declare that the data supporting the findings of this study are available within the paper. Should any raw data files be needed in another format they are available from the corresponding author upon reasonable request.
